# Effects of forest areas on air quality; Aras Basin and its environment

**DOI:** 10.1186/2052-336X-12-60

**Published:** 2014-03-10

**Authors:** Metin Demir, Turgay Dindaroğlu, Sevgi Yılmaz

**Affiliations:** 1 Department of Landscape Architecture, Architecture and Design Faculty, Ataturk University, 25240 Erzurum, Turkey; 2 Department of Forest, Sütçüimam University, 46100 Kahramanmaraş, Turkey

**Keywords:** Air quality, Air control, Forest land, Aras Basin, Planning

## Abstract

**Background:**

In the study, the Aras Basin and its environment, one of the most important hydrological basins of Turkey, was evaluated. In survey area, to determine the change of air quality, it was benefited from 23,770 pieces of hourly measured SO_2_ (Sulfur dioxide) and PM_10_ (particulate matter) concentration values for the December, January and February of 2009–2010 in which the pollution is at peak, by forming database in geographical information system (GIS), spatial analyze maps were attained. By comparing; maps showing attained numeral air quality and maps showing the spread of forest lands in the region, it was tried to determine the relation and interaction between air quality and forest lands.

**Results:**

The results indicated that the Air Quality Index (AQI) values were the lowest for the forest land in the months which mean that the forest land was the most convenient place for health. The increase the AQI, air pollution also increases. The results indicated that the air quality index changed from 1 to 4 within the region. In the forest areas, the AQI values for the months were the lowest. This indicated that the most suitable places for health are the places with a high forest coverage rates (76,50; 66,46 and 96,78%). There was no forest area within the region where the AQI values were the highest, so the risk was maximum, for the months.

**Conclusions:**

Authorities should create new afforestation areas and rehabilitate degraded forest lands to limit air pollution by increasing the quality of urban life.

## Introduction

Forest land is equal to 31% of the territory in the world. More than 1,5 billion people get living from forests. Besides countless benefits, forests have great importance in sustainability of important ecosystem functions as protecting biological variation, protecting earth, water quality and supply, flood control, climate adjustment and recreation [1,2].

Forests are one of the most important ecosystems affecting earth climate. They carry out important functions enhancing air quality. They prevent pollution of the cities by sticking particle and aerosols on leaf surface, absorbing and by slowing down the air movements make them fall into surface. Also, they reduce greenhouse gas effect by avoiding CO_2_. In many surveys conducted, it was shown that a green zone surrounding a city decreases the lead percentage approximately 85% [3].

As a result of developments on urbanization and especially increase in the city populations, air pollution is the leading environmental problem affecting human health and decreasing quality of life in especially cities.

Air pollution can be defined as being pollutants as sulfur dioxide, particulate matter, nitrogen oxide, and ozone in such a level that have negative effects on environment and health [4-9].

Among the main causes of air pollution in our country, industrial facilities, fossil fuels and exhaust gases are the most important. However the main reason triggering these causes, uncontrolled population increase and concordantly, urbanization and destroying or exterminating green spaces can be counted [10,11].

For this reason, it is required to make air quality management plans, have valid and reliable information about existing pollution for a rationale planning. For this purpose, level of pollutant concentration must be measured in several time intervals, and it has to be known the reliability of these measurements.

It is very important observing air quality to determine the sources of air pollution and topographical distribution, to develop control strategies, and to control the effectiveness of these basic air pollutants. Possible to be found in urban areas can be counted as carbon monoxide (CO), sulfur dioxide (SO_2_), particulate matter (PM), ozone (O_3_), nitrogen dioxide (NO_2_), and lead (Pb). Negative risks that can be caused by these pollutants are also related to many factors as meteorological and physiographic features as well as uncontrolled industrialization, population growth and transportation [12].

For observation of air pollution data, spatial analyzes and modeling, observation, analyze, modeling and showing on the map functions of the geographical information systems (GIS) can be used [13-17].

In this study, it is aimed to analyze relation and interaction between air quality and forest lands in Aras Basin and its environment. For this purpose, it is benefited from sulfur dioxide (SO_2_) and particulate matter (PM_10_) values while forming Air Quality Index maps in GIS. Air Quality Index maps are evaluated by comparing numerical Forest management maps showing present forest land and its distribution in the region.

## Materials and methods

**Figure 1:**
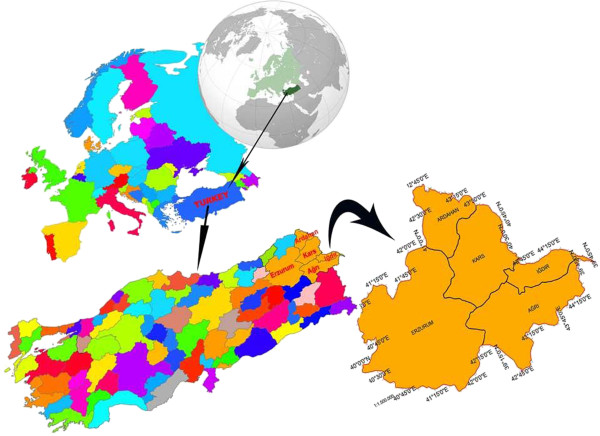
Research area “Aras Basin and its environment”.

**Figure 2:**
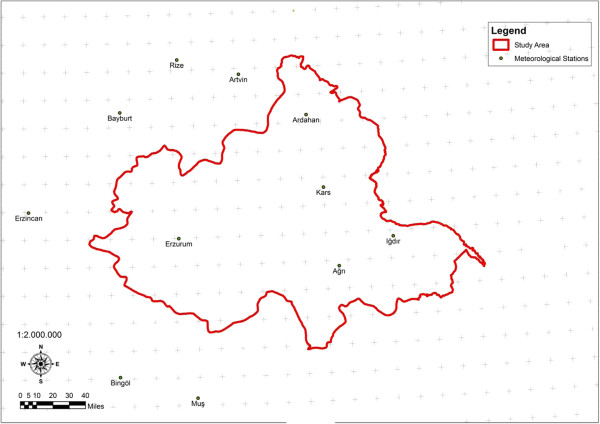
Air quality index measurement station locations.

**Figure 3:**
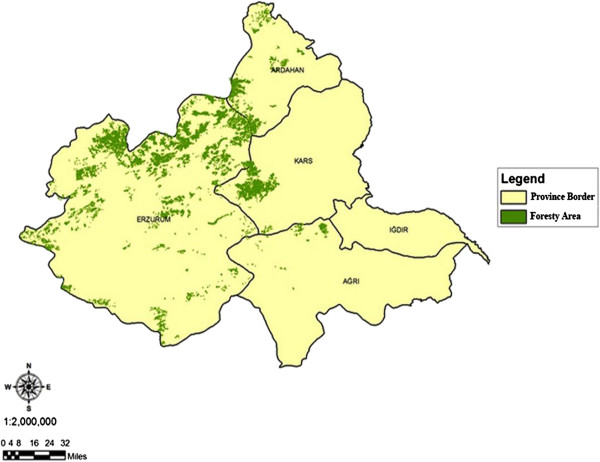
Forest areas of research area.

The main material of the research is formed by one of the important hydrological basin Aras Basin (Figure 1), forest management plans showing interior natural forest existence and Air Quality Index map (AQI) values of inside the basin borders and Erzurum, Erzincan, Ardahan, Iğdır, Kars, Ağrı, Bingöl, Muş, Bayburt, Rize cities in its environment. In Figure 2, the location of the 11 stations of which measurement values used, in Figure 3, the numerical maps showing forest existence in these lands are given. Important rivers of the region are Aras and Kura rivers [18] (EAP 2010). Research area is 54.605 km^2^ and it is between 40^º^02′0′′ and 39^º^45′0′′ north latitudes and 40^º^30′0′′ and 44^º^15′0′′ east longitudes.

In this study, numerical forest management maps showing present forest lands in the survey borders and inside the survey area and its environment, Air Quality Index values of 11 cities of 2009–2010 years December, January and February months were analyzed. In the survey area, changes in air quality because of heating in winter months are seen. The most important cause of the air pollution in the region is fossil fuels used in residence and industrial areas in winter months. As a result, in the study, the AQI of dense heating periods; December, January and February are used.

Air Quality Index (AQI) is an index used for reporting daily the air quality of the environment where we live. It gives information about how clean or polluted the air of the area where we live and what health effects it may cause.

The AQI give information about effects that may appear after a couple of hours or a couple of days after polluted air inhaled. The AQI is a scale formed in the range of 0–500 (Table 1). As The AQI increases, air pollution increases and directly or indirectly some health problems arises [19]. The AQI scale can be examined in 6 categories [20].

**Table 1:** Air quality index scales [20]

**AQI scale value**	**Air Quality Index (AQI)**	**Health level**
1	0-50	Good
2	51-100	Middle
3	101-150	Unhealthy for sensitive groups
4	151-200	Unhealthy
5	201-300	Very unhealthy
6	301-500	Dangerous

The AQI values were calculated separately for every pollutant in the region (ozone, particles, carbon monoxide, sulfur dioxide, and nitrogen dioxide). Maximum calculated the AQI value, forms the the AQI value of the day [12].

In the study, Geographical Information System (GIS) is used as a means. Firstly, for measurement stations, spot database is formed by means of ArcGIS 9.3 software according to national coordinate system (UTM-37 Zone). Forming maps showing the distribution of AQI and pollutant value onto the region best, “Kriking” interpolation in the Spatial analyst extension tab of ArcGIS 9.3 software of ESRI Firms used. Using maps based on this technique, spatial analyzes realized and, distribution of SO_2_ and PM_10_ amounts were determined. By Kriking method, making use of data present in the study field experimental variogram model is formed. By determining the optimal model, areal transformation was carried out. Kriking formula related to the model is given below [21].

$$\begin{array}{l} Z_{p}=\sum_{i=1}^{n}W_{i}Z_{i}\\ \mathrm{Here};\;\mathrm{Zp}:\;\mathrm{expected}\;\mathrm{undulation}\;\mathrm{value}\;\mathrm{of}\;P\;\mathrm{point}.\\ \mathrm{Wi}:\;\mathrm{weight}\;\mathrm{values}\;\mathrm{corresponding}\;\mathrm{to}\;\mathrm{every}\;\mathrm{Zi}\\ \mathrm{Zi}:\;\mathrm{undulation}\;\mathrm{value}\;\mathrm{of}\;\mathrm{used}\;\mathrm{spots}\\ n:\;\mathrm{number}\;\mathrm{of}\;\mathrm{spots}\;\mathrm{used}. \end{array}$$

Involve of the areas that has no station values in survey area in to the analyses analyzes, can be possible by transferring spatial AQI values of the stations near environment of the survey area with Kriking interpolation technique into GIS. While creating air quality maps “Geostatistical Analyst” module was used and different models were evaluated on ArcGIS 9.3 software.

While evaluating models, mean error (ME) must be close to 0 and approximate standardized mean errors’ square root (RMSSE) close to 1 [22]. While implementing the formulas, it is surveyed whether there is anisotropy effect or not. Also to evaluate the AQI values of the areas where there is no station data, the measurement results of neighbor areas; Rize, Artvin, Bayburt, Erzincan, Muş and Bingöl were used (Table 2).

**Table 2:** Location and time of observations at air quality measurement stations

**No**	**Air quality measure station**	**Coordinates (Latitude-Longitude)**	**Altitude (m)**	**Observation months (2009–2010)**
**1**	AĞRI	39.722222-43.405000	1632	December-January-February
**2**	ERZURUM	39.898056-41.272500	1950	December-January-February
**3**	ARDAHAN	41.110833-42.701111	1810	December-January-February
**4**	IĞDIR	39.926111-44.053611	865	December-January-February
**5**	KARS	40.605000-43.104444	1758	December-January-February
**6**	MUŞ	38.748611-41.509444	1312	December-January-February
**7**	BİNGÖL	38.883889-40.503611	1140	December-January-February
**8**	ERZİNCAN	39.743056-39.495000	1193	December-January-February
**9**	BAYBURT	40.258889-40.224167	1568	December-January-February
**10**	ARTVİN	41.175278-41.818333	628	December-January-February
**11**	RİZE	41.021944-40.532500	21	December-January-February

While forming database of the AOI values, in 11 cities 23770 pieces of hourly sulfur dioxide (SO_2_) and Particulate Matter (PM) measurement results of ministry of environment and urban planning inside Aras Basin were evaluated (Table 3) [23].

**Table 3:** **(SO**_**2**_**) and (PM**_**10**_**) measurements attained in survey area**

**Number no**	**Date**	**Station**	**SO**_**2**_	**PM 10**	**MAX**	**Max Name**
**1**	12.01.2009 00:00	AGRI	1	2	2	PM 10
**2**	12.01.2009 01:00	AGRI	1	2	2	PM 10
**3**	12.01.2009 02:00	AGRI	2	2	2	SO2
**4**	12.01.2009 03:00	AGRI	1	2	2	PM 10
**..**						
**…**						
**….**						
**23767**	2/28/2010 9:00:00 PM	RIZE	1	2	2	PM 10
**23768**	2/28/2010 10:00:00 PM	RIZE	1	2	2	PM 10
**23769**	2/28/2010 11:00:00 PM	RIZE	1	2	2	PM 10
**23770**	03.01.2010 00:00	RIZE	1	2	2	PM 10

In the study, to determine the effect of forest lands on air quality, AQI maps formed on GIS are compared with numerical forest management maps showing the present forest areas inside the survey area and the distribution, and the interaction and relation between air quality and forest area are tried to be evaluated.

## Results

The study area was the Aras Basin which include Erzurum, Ardahan, Iğdır, Kars, and Ağrı cities, and total area is 227.951 km^2^. According to [24] report, when we think of the water shortage the world will go through and the climate change, importance of this survey will be seen better in terms of pointing out basins.

Data attained from the 11measurement stations, reveal that there are differences in air quality in winter months; in terms of months and distribution of area.

For air quality values in the survey area did not show directional change, therefore isotropic semivariogram model was preferred. Spatial dependency (Nugget/Sill ratio) is about autocorrelation degree of sampling points. If spatial dependency is high, spatial correlation between sampling points are so high. Spatial dependency (%177 and %153) found as a low between sampling points out of the distributions of meteorological stations are not enough. Effective range where spatial dependency active are 271470 meters (Table 4). Kriking interpolation that is used for converting spot data to areal, “Exponential” model has low failure rate for SO_2_. Other hand Gaussian model has more spatial correlation with PM_10_. Map suitable for this model was formed and areal data was attained from this map.

**Table 4:** Variogram models and model parameters of attained spatial maps regarding air quality

**Pollutants type**	**Model**	**Nugget**	**Range**	**Sill**	**(Nugget/Sill) %**	**ME**^**1**^	**RMSSE**^**2**^
SO_2_	Exponential	0,0479	271470	0,0270	177,41	0,2965	0,8813
PM_10_	Gaussian	0,3920	271470	0,2560	153,13	0,8223	0,8577

ME1: standard error of the mean.

RMSSE2: estimated standardized mean of error of mean square root.

**Figure 4:**
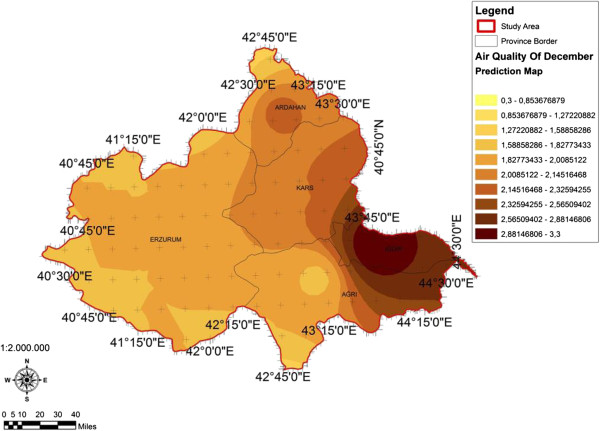
Spatial analyze map of 2009-2010 year December month air quality concentrations.

**Figure 5:**
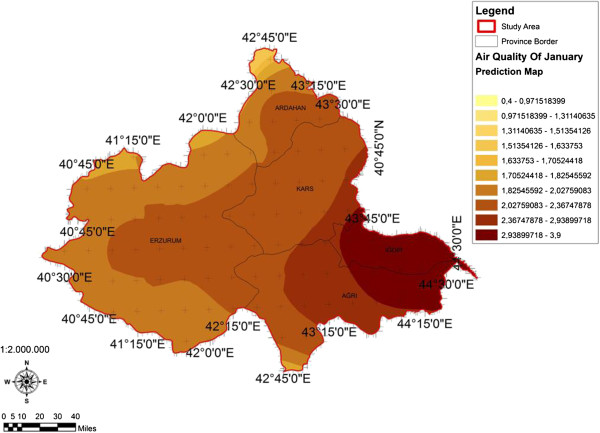
Spatial analyze map of 2009-2010 year January month air quality concentrations.

**Figure 6:**
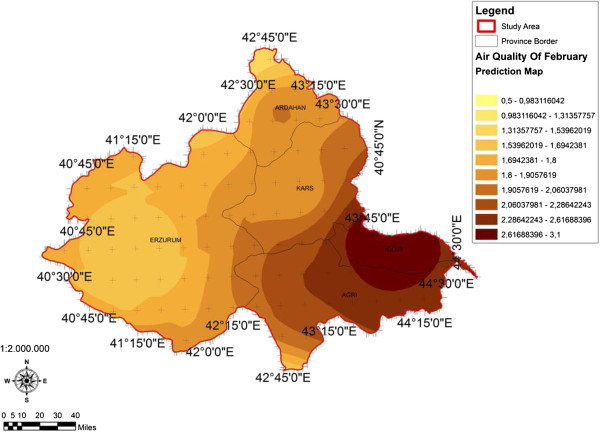
Spatial analyze map of 2009-2010 year February month air quality concentrations.

**Figure 7:**
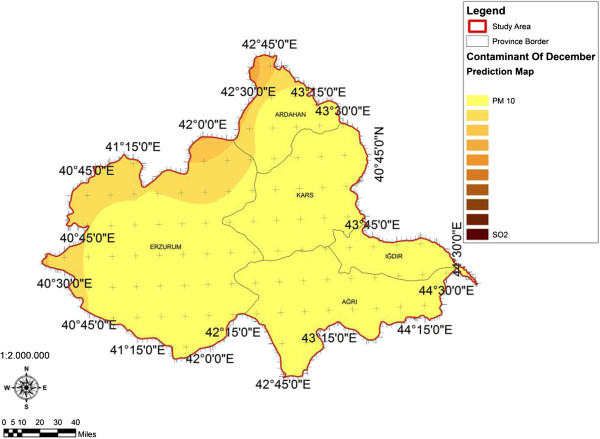
**Spatial analyze map of 2009-2010 year December month SO**_**2 **_**and particulate matter concentrations.**

**Figure 8:**
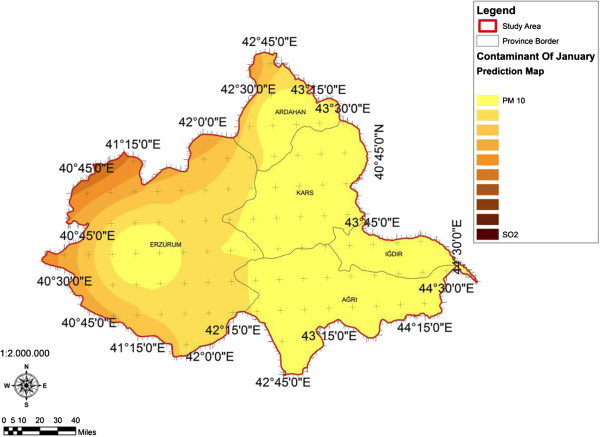
**Spatial analyze map of 2009-2010 year January month SO**_**2 **_**and particulate matter concentrations.**

**Figure 9:**
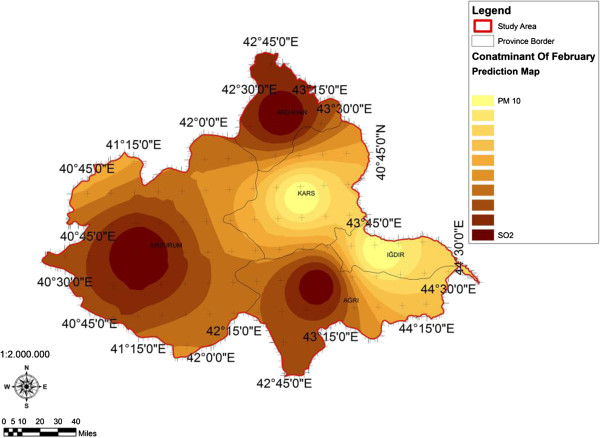
**Spatial analyze map of 2009-2010 year February month SO**_**2 **_**and particulate matter concentrations.**

When the AQI values for Decemcer, January and February of 2009–2010, it would be seen that the AQI scale gets values between 1 and 4. It was determined that Ağrı was the region which is exposed to the densest pollution in December (Figure 4). In this region the AQI value changes between 2.0-3.3 range and poses a risk for sensitive groups. However in January, it was determined that the AQI values changes between 2.0-3.9 and risky regions for sensitive groups were Iğdır, Ağrı, Kars, Erzurum and Ardahan (Figure 5). However in February, it gives the results that the AQI values changes between 2.0-3.1 range and this change affect spatially Iğdır and Ağrı cities (Figure 6). When the concentrations of pollutants in December, January and February were analyzed, in December in most of the survey area high concentrated pollutant PM10 is found (Figure 7). In January, high concentrated pollutant PM_10_ was less than December and (SO_2_) concentration intensifies to the west of Erzurum (Figure 8). Also in February, there is a possibility of the population to be exposed to pollution, and the areas where the highest SO2 concentrations seen were: Erzurum, Ardahan, and Ağrı city centers (Figure 9).

**Figure 10:**
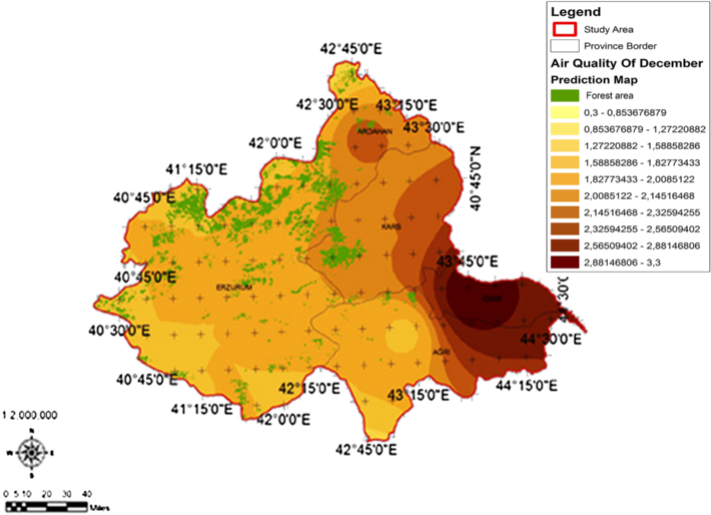
2009-2010 year December month spatial analyze map of the relation between forest area and air quality.

**Figure 11:**
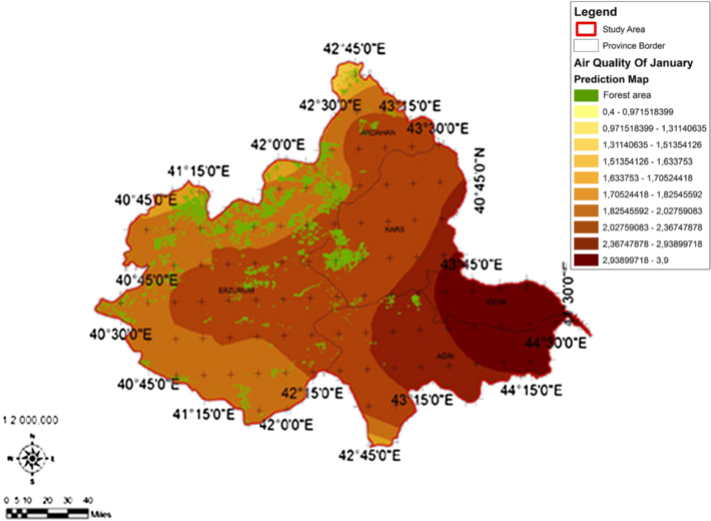
2009-2010 year January month spatial analyze map of the relation between forest area and air quality.

**Figure 12:**
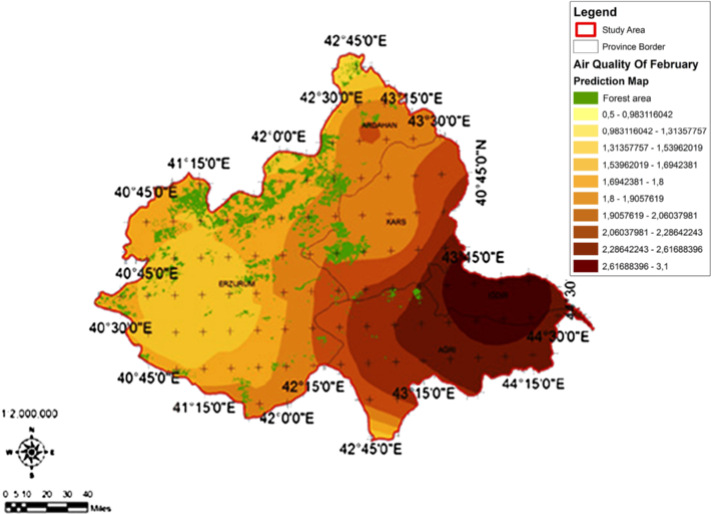
2009-2010 year February month spatial analyze map of the relation between forest area and air quality.

When we evaluate forest lands according to months and areas depending on the AQI values in survey basin, the areas of which AQI values in December was the lowest and the most appropriate for health, contains %76,50 forest lands which was the highest ratio of all (Figure 10). In January, in areas where the AQI is the lowest, forest lands have the greatest area of % 66, 46 (Figure 11). In February, it has been understood that in areas where the AQI values were the lowest, forest lands had a great ratio as % 96,78 (Figure 12). It determined that in all of December, January and February months, the AQI values were the highest, in other words there was no forest land in places where the risk was the highest (Table 5).

**Table 5:** Air quality and spatial distribution of forest areas

**Month**	**Air quality index value**	**General area**		**Forest area**	
		**Km**^**2**^	**%**	**Km**^**2**^	**%**
**December**	2	32145.26	58.87	2342.13	76.50
	3	21187.97	38.80	719.64	23.50
	4	1272.66	2.33	0.00	0.00
	**Total**	54605.89	100.00	3061.77	100
**January**	2	15436.92	28.27	2035	66.46
	3	33598.32	61.53	1026.77	33.54
	4	5570.64	10.20	0.00	0.00
	**Total**	54605.89	100.00	3061.77	100
**February**	2	38197.10	69.95	2963.22	96.78
	3	15873.86	29.07	98.55	3.22
	4	534.93	0.98	0.00	0.00
	**Total**	54605.89	100.00	3061.77	100.00

## Discussions

To protect habitats, climatic and natural sources as soil, water and air must be kept out of negative pressures. Despite controlling air pollution levels regularly, negative effects range over limit values. The reason is that the increase especially in industrial facilities, using low quality heating fuels and vehicles exhaust gases. Rapid urbanization which causes all these and decrease of green land which tolerate these causes, show that who important to handle this problem [25-30].

When we analyze the information attained from this survey, air quality index gets values between 1 and 4 and it is seen that these values are above WHO values. Iğdır city, which has the highest values that is in the first place unhealthy for sensitive groups, is on the one hand surrounded by Ağrı mountain chain, on the other hand being a plain which brings a hollow terrain structure, has an area which blocks airflow.

Because of this terrain structure, Iğdır experience serious air pollution problem in winter months. Parallel to survey results, according to Turkish Statistical Institute air pollution values, Iğdır is the city which has the highest mean particulate matter ratio. Iğdır has the first place according to pollution row [31].

Though natural effects has little effect ration on air pollution, the real effect is human activities. This bad condition in air quality can be explained with solid and liquid fuels which do not meet standards, locally burning materials as cow dung which billows, air pollution resulting from traffic and inadequate controlling. Not reaching of natural gas attained from Iran to Iğdır and Ağrı, restrains air quality in these cities reach to desired WHO levels. It seems that making the air quality in Iğdır is bound to using natural gas and enhancing controls in the city.

Erzurum, Kars, Ağrı and Ardahan cities, are in a area, with their topographic and geographic structure, which lives through continental climate. In these cities, which have the lowest mean temperature degrees, winters are very harsh and cold. From the results attained, the most notably one is that in February, Kars has the most particulate matter ratio than Erzurum, Kars and Ardahan despite the fact that they have the same continental climate conditions. It is seen that because of using fossil fuels for heating, also because of socio-economic conditions using cheap but low calorie and high sulfur ratio coals much, and because of bad meteorological conditions which needs high energy for heating, air pollution come to a position which need to be taken precautions.

As seen from the spatial analyze maps prepared according to geographical information system (GIS) measurement values, parallel to results of many studies proving the relation between air quality and forest lands, forest land affect air quality positively. Regarding survey results attained form comparing spatial distribution of the AQI bound to time with forest land existence in the area, the AQI values is low in areas where forest land density is high, and high in areas where forest land density is low, it reveals the result that how forest lands are important on the AQI values. When we analyze forest lands according to months and areas dependent to AQI values in survey area; it was determined that the AQI was low in December, January and February, in other words, in areas which are the most appropriate for health, forest coverage rates are the highest with %76,50, %66,46 and %96,78. It stands out that in areas where the AQI has the highest value in December, January and February, in other words, where there is the highest risk, there are no forest lands. Thus, there have been many surveys conducted supporting this survey result. It was determined [32], that 6,000,000 trees absorb 9,000 tons particulate matter and 304,000 tons carbon dioxide. Forest ecosystems, which are one of the most important of continental ecosystems, absorb approximately 100 gigatonne (Gt, Billion ton) CO_2_ from atmosphere, and release half of it back. It reveals the importance of forest ecosystems on this matter [33]. As sulfur dioxide and other pollutants, released from different sources, are absorbed by plant tissues; this provides air to clean and thus air quality to increase [34-39]. Accumulation of CO_2_ in the atmosphere arises from using fossil fuels and deforestation caused by misuse of areas [40].

## Conclusions

As a conclusion, to limit air pollution, one of the most important environmental problems of recent days; we should raise air quality standards, people should be warned via early warning systems, and source control mechanisms (emission control) must be improved. Besides countless benefits, having many function on increasing air quality, new afforestation areas must be created and degraded forest lands must be rehabilitated.

## Competing interests

The authors declare that they have no competing interests.

## Authors’ contributions

MD and TD participated in the design of the study. MD have collected the data and drafted the manuscript with SY. TD used the GIS system and make statistical analysis. MD and SY related data with forest area. All authors read and approved the final manuscript.
